# Single‐Neuron Critical Intermittency in a Stochastic Hodgkin–Huxley Model

**DOI:** 10.1111/ejn.70355

**Published:** 2025-12-17

**Authors:** Konstantinos Varvaras, Fotios K. Diakonos, Efstratios K. Kosmidis

**Affiliations:** ^1^ Department of Medicine, Laboratory of Physiology Aristotle University of Thessaloniki Thessaloniki Greece; ^2^ Department of Physics University of Athens Athens Greece

**Keywords:** complex systems, ion channels, membrane potential, power‐law, self‐organization

## Abstract

Brain criticality has emerged as a rapidly growing focus of research among neuroscientists and physicists. The latest experimental evidence suggests that even isolated neurons display signs of criticality. Using a stochastic type‐I parametrization of the Hodgkin–Huxley model, we investigate the origin of these critical dynamics. We show that the model adequately approximates the experimentally observed behavior, as it reproduces the qualitative relationship between the critical state and both the applied external stimulation and the spiking rate. External white noise further enhances any pre‐existing critical intermittency but cannot by itself toggle the system into a critical state. The emergence of the critical state is conditional on the system's proximity to its spiking bifurcation point, and any divergence from it results in the abolition of the dynamics. We treat the neuronal membrane as a complex self‐organizing system composed of interacting ion channels and propose that the observed dynamics result from an *almost critical* state.

## Graphical abstract

We implemented a stochastic Markovian Hodgkin–Huxley model to simulate the function of the neuronal membrane. The resulting membrane potential time series were analyzed using the Method of Critical Fluctuations and were found to exhibit critical intermittency, which was conditional to the system's proximity to its spiking threshold. These findings are consistent with the behavior observed in in vitro neurons.

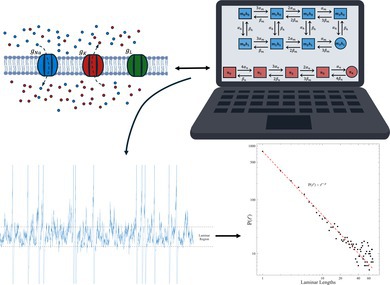

**Abbreviations**
MCF: Method of Critical Fluctuations
SOC: self‐organized criticality
SOqC: self‐organized quasi‐criticality

## 1 Introduction

One can argue that nature is neither restricted in a fully ordered monotonous state, nor a chaotic unpredictable system where future predictions would be impossible. What makes the universe interesting to us is its inherent complexity. In the last few decades, in an attempt to study and explain the emergence of complexity from seemingly simple and deterministic physical laws, scientists have put aside the reductionist angle and instead adopted a statistical mechanics top‐down approach. This venture gave birth to the idea of self‐organized criticality (SOC) as a possible mechanism for the spontaneous emergence of complexity in natural dynamical systems (Bak et al. 1988; Bak 1996). Ever since, a lot of natural phenomena, from earthquakes (Carlson and Langer 1989) and forest fires (Clar et al. 1999) all the way up to biological evolution (Kauffman and Johnsen 1991; Sneppen et al. 1995) and epidemics (Stollenwerk 2005), have successfully been studied and interpreted from this viewpoint.

SOC theory suggests that dynamical systems composed of many interacting elements with numerous degrees of freedom and non‐linear dynamics tend to spontaneously evolve in a transitional state, between order and chaos, the so‐called “critical state”. They seem to have an attractor right at, or in the neighborhood of a second‐order phase transition. This mode of operation is characterized by universality between drastically different systems and scale‐freeness, which gives rise to spatiotemporal power‐law scaling and fractal patterns (Bak 1996; Christensen and Moloney 2005; Marković and Gros 2014).

In this context, neuroscientists and physicists have put forward the “Critical Brain Hypothesis”, which views the brain as an ensemble of trillions of neurons whose interactions form a complex system exhibiting critical dynamics (Stassinopoulos and Bak 1995; Herz and Hopfield 1995; Chialvo 2004; Cocchi et al. 2017). Criticality in the brain is appealing, as it can account for its ability to efficiently process the world. Systems operating at the critical point manifest among others optimal dynamic range (Kinouchi and Copelli 2006; Shew et al. 2009), peak information capacity, transmission, and memory (Haldeman and Beggs 2005; Shew et al. 2011). Furthermore, it could also provide a possible explanation for the evolution and development of such a complex structure through a process of SOC (Bak 1996).

Since the conception of the critical brain hypothesis, numerous studies have reported signs of criticality in various scales and contexts. The landmark experiments of Beggs and Plenz in both in vivo and in vitro networks of neurons (Beggs and Plenz 2003, 2004) reveal a power‐law scaling in the distribution of local field potential events, which they famously named neuronal avalanches. Subsequent studies in rat and monkey brains verified their results (Petermann et al. 2009; Ribeiro et al. 2010). Additionally, whole brain studies in humans show a scale‐free distribution of activity clusters in fMRI signal (Tagliazucchi et al. 2012) and power‐law scaling in both space and time in EEG and MEG signals (Linkenkaer‐Hansen et al. 2001; Shriki et al. 2013).

Although single neuronal cells were first viewed as mere simple components of a complex system, accumulating evidence points to the existence of criticality in the single‐neuron level. Chemically isolated mammalian neurons have been shown to exhibit slower recovery from perturbations as they approach their spiking threshold, a phenomenon called “critical slowing down” (Meisel et al. 2015). In addition, neuronal excitability seems to manifest intermittency and timescale invariance, in a manner not correlated with the behavior of neighboring neuron cells, but rather arising from the intra‐neuron dynamics (Gal et al. 2010). These results were also sufficiently reproduced by biophysical models of single neurons (Steyn‐Ross et al. 2006; Soudry and Meir 2012; Gal and Marom 2013; Bukoski et al. 2015), suggesting that the ensemble of ion channels of the neuronal membrane is capable of giving rise to complex dynamics. Much like the whole brain, criticality at the cellular level could provide computational advantages and enhance inter‐neuron information transfer (Meisel et al. 2015).

In our previous work, we demonstrated that the dynamics of sub‐threshold membrane potential fluctuations of rat hippocampal pyramidal neurons exhibit intermittency and suggest proximity to a critical point. We also suggested that this experimental finding is the imprint of the inter‐channel interactions of the neuronal membrane, suggesting single‐neuron criticality. This behavior was not reproduced by simply adding white noise to a deterministic Hodgkin–Huxley model (Kosmidis et al. 2018).

Here, we examine if a stochastic version of the Hodgkin–Huxley model is able to reproduce the intermittent dynamics observed in our in vitro experiment. We show that the simulated time series reproduce qualitatively most of the experimental findings. We further explore the underlying generative mechanism of the dynamics, and we suggest a self‐organization into an almost critical state when the system is in the neighborhood of the spiking bifurcation.

## 2 Materials and Methods

### 2.1 Method of Critical Fluctuations

The simulated timeseries were analyzed using the Method of Critical Fluctuations (MCF) (Contoyiannis et al. 2002). The algorithm is based on the idea that the fluctuations of the order parameter Φ of a system exhibiting critical dynamics can be described by a 1‐d nonlinear map (Contoyiannis and Diakonos 2000), which can be approximated as follows:

(1) $$\Phi_{n+1}=\Phi_{n}+{\mathrm{u\Phi}}_{n}^{z}+\varepsilon$$

where $\Phi_{n}$ is the value of the scaled order parameter at the timestep n, u is a coupling parameter, z is associated with the isothermal critical exponent and ε is a noise term, accounting for the finite size of the system (Contoyiannis et al. 2002, 2004).

The invariant density $\rho \left(\Phi \right)$ of the chaotic trajectories resulting from the Critical Map (1) is characterized by a plateau region, where fully correlated dynamics take place, namely the laminar region. We denote the starting and the ending point of the laminar region as $\Phi_{0}$ and $\Phi_{R}$ respectively. The time intervals spent in this region are called laminar lengths (ℓ). The kth laminar length $\ell_{k}$ is defined as the number of successive values of Φ fulfilling the condition $\Phi_{0}\leq \Phi_{i}\leq \Phi_{R},\;i=k+1,k+2,\ldots ,k+\ell$. The laminar lengths have a characteristic power‐law distribution:

(2) $$P\left(\ell \right)\sim \ell^{-p}$$

where $p=\frac{z}{z-1}$. For a thermal critical system p>1 (Contoyiannis and Diakonos 2007).

This invariant density $\rho \left(\Phi \right)$ is equivalent to the distribution of the order parameter of a system operating at the critical point.

**FIGURE 1:**
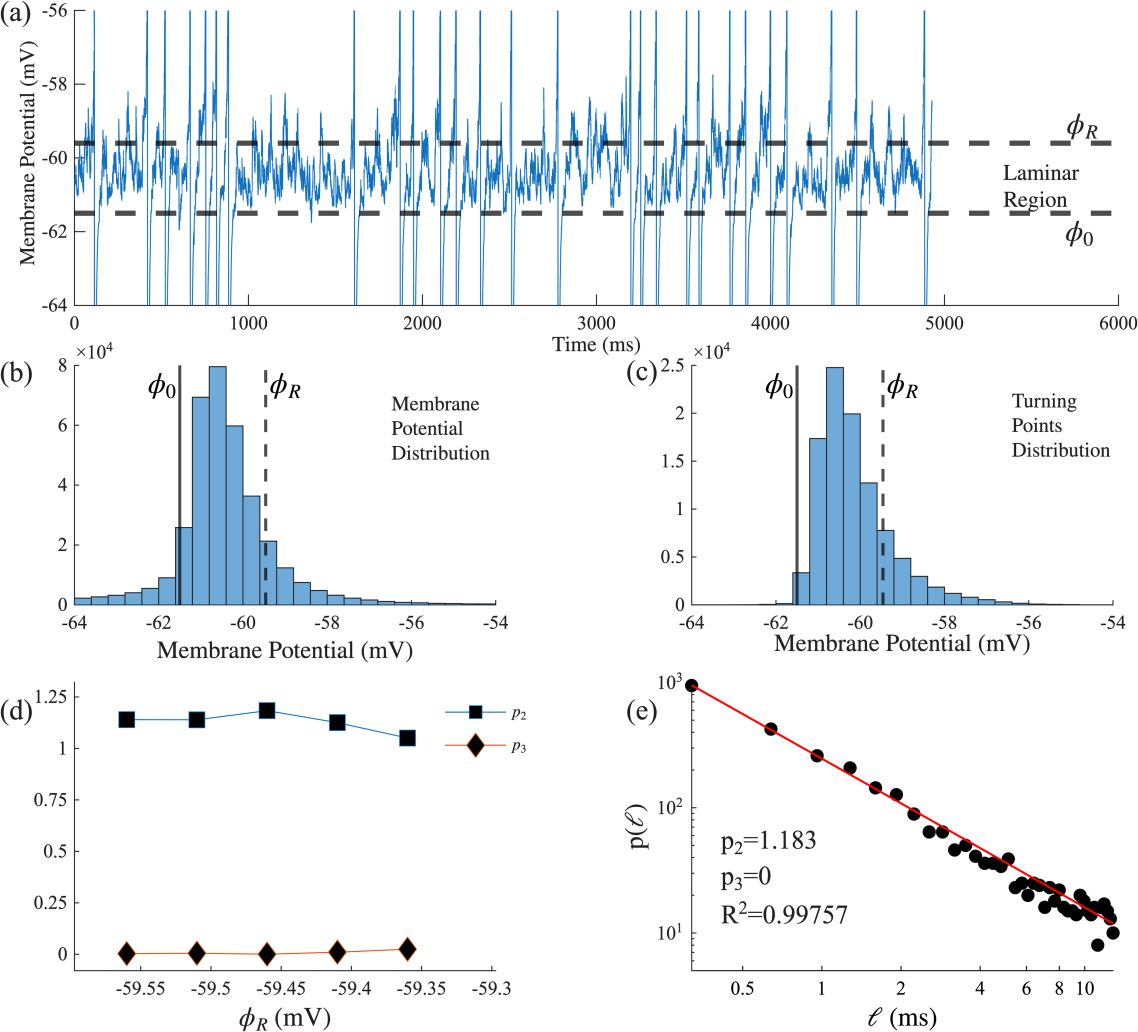
(a) A segment of a simulated time series with $I_{\mathrm{ex}}=0.35\;\mathrm{\mu A}/{\mathrm{cm}}^{2}$ *.* The horizontal dashed lines indicate the laminar region. (b) Distribution of the membrane potential of the time series. (c) Distribution of the turning points of the time series. (d) $p_{2}$ and $p_{3}$ exponents as a function of the laminar region end point $\phi_{R}$, for $\phi_{0}=-61.5$ *mV*. (e) The distribution of laminar lengths $\left(\ell \right)$ for $\phi_{R}=-59.46\;\mathrm{mV}$.

Based on this groundwork, we can apply the algorithm in a timeseries of the neuron membrane potential, assuming the existence of a fixed point and viewing the membrane potential V as the system's order parameter *Φ* (Kosmidis et al. 2018). We can roughly estimate the location of the starting point of the laminar region $\Phi_{0}$ as the fixed point of the time series (Contoyiannis et al. 2004). This is accomplished using the histogram of the turning points. A turning point of a trajectory of the map (1) is defined as a local maximum or minimum of the trajectory. In the same way, we can define the turning points of a time series as local extrema points of the time series. The existence of fixed points leads to discontinuities in the turning points distribution (Diakonos and Schmelcher 1997). Figure 1c depicts the distribution of the turning points of a membrane potential time series, where the abrupt left side of the distribution indicates the existence of a fixed point around that area and thus the position of $\Phi_{0}$. Theoretically, the ending of the laminar region is located at the point where the non‐linear term of the map (1) (${\mathrm{u\Phi}}_{n}^{z}$) becomes significant. In practice, we cannot determine the exact value of $\Phi_{R}$ and we treat it as a variable in our analysis. For a number of different equally spaced end points $\Phi_{R}$, we calculate the distribution of the laminar lengths $P\left(\ell \right)$ as a function of $\Phi_{R}$. We then fit the obtained distributions to the function:

(3) $$f\;\left(\ell \right)=p_{1}\ell^{-p_{2}}e^{-\ell p_{3}}$$

to obtain the exponents $p_{2}$ and $p_{3}$, which have a competitive role and their exact values depend on the choice of $\Phi_{R}$. Lastly, we plot the aforementioned exponents as a function of $\Phi_{R}$. The function $f\;\left(\ell \right)$ approaches Equation (2) as $p_{3}\to 0$. Criticality is therefore detected when $p_{2}>1$ and $p_{3}\approx 0$ for a range of $\Phi_{R}$ values. We repeat this process for an increasing number of end points, ideally covering the whole range of fluctuations' amplitude, until the best power‐law distribution of laminar lengths is found. The minimization of $p_{3}$ is used as a criterion to estimate the actual value of $\Phi_{R}$. This process must be repeated for each time series, leading to unique limits of the laminar region that cannot be used for a different one. Note that the limits of the laminar region are not strict, but that does not affect the results of our analysis.

Based on the sampling rate of the time series, we have transformed the laminar length units, from a dimensionless number of time steps to time units.

Curve fitting was performed in MATLAB R2021a with the non‐linear least squares optimization using the trust‐region‐reflective algorithm and $R^{2}$ was used as a goodness of fit measure.

### 2.2 Stochastic Hodgkin–Huxley Integrator

The model implemented here is based on the golden standard introduced by Hodgkin and Huxley (Hodgkin and Huxley 1952). In their approach, the dynamics of the neuronal membrane are described by the following differential equation:

(4) $$C_{m}\frac{\mathrm{dV}}{\mathrm{dt}}={\bar{g}}_{\mathrm{Na}}m^{3}h\left(E_{\mathrm{Na}}-V\;\right)+{\bar{g}}_{K}n^{4}\left(E_{K}-V\;\right)+g_{L}\left(E_{L}-V\;\right)+I_{\mathrm{ex}}$$

where V is the transmembrane potential in respect to the extracellular environment, ${\bar{g}}_{\mathrm{Na}}$ and ${\bar{g}}_{K}$ are the maximum sodium and potassium conductances, $g_{L}$ is the leakage ion channel conductance, $E_{\mathrm{Na}}$, $E_{K}$, and $E_{L}$ are the sodium, potassium and leakage ion channel reversal potentials respectively, $I_{\mathrm{ex}}$ is the external current stimulation.

n, m, and h are the gating variables describing the potassium channel activation, sodium channel activation and deactivation respectively. Each one of them is described by the following equation:

(5) $$\frac{\mathrm{dx}}{\mathrm{dt}}=\alpha_{x}\left(V\right)\left(1-x\right)-\beta_{x}\left(V\right)x,x=m,n,h$$

with $\alpha_{x}\left(V\right)$ and $\beta_{x}\left(V\right)$ being the voltage‐dependent rate constants for gate opening and closing, respectively.

The inability of the original deterministic Hodgkin–Huxley model to reproduce our experimental findings, despite the addition of white noise (Kosmidis et al. 2018), lead us to implement a Markovian stochastic model (Fox and Lu 1994; Chow and White 1996). In contrast to the original mean‐field approach, one could consider the effect of the independent channel gates to be significant in the single channel level. Each sodium channel is composed of 3 m and 1 h gate, while each potassium channel of 4 n gates. A channel only conducts when all of its gates are in the “open” state. The transition of the “open” state to the “closed” one and vice versa is inherently probabilistic and is determined by a simple Markov chain process:

(6) n04αn⇌βnn13αn⇌2βnn22αn⇌3βnn3αn⇌4βnn4

(7) m0h03αm⇌βmm1h02αm⇌2βmm2h0αm⇌3βmm3h0αh⇃↾βhαh⇃↾βhαh⇃↾βhαh⇃↾βhm0h13αm⇌βmm1h12αm⇌2βmm2h1αm⇌3βmm3h1

where the indices on n, m, and h correspond to the number of open gates of each type. This scheme introduces 13 different channel states, 8 for Na^+^ and 5 for K^+^, and 28 possible state transitions, while $n_{4}$ and $m_{3}h_{1}$ correspond to the conductive potassium and sodium channel states respectively.

In this paper, the parameterization of the Hodgkin–Huxley model is based on Nowotny and Rabinovich (Nowotny and Rabinovich 2007), which describes a type‐I behavior, according to Hodgkin's classification (Hodgkin 1948). This choice was made considering two main reasons. First, this type of model was built upon a mammalian hippocampal cell model (Traub and Miles 1991) matching the origin of our in vitro acquired data. In contrast, the original Hodgkin–Huxley parameterization is a phenomenological model based on the study of the squid‐giant axon. Second, the shape, patterns and power spectrum of the membrane potential fluctuations of the in vitro recordings best match an integrator‐type model, rather than a resonator one. Scaling the parameters to a standard specific capacitance C of 1 $\mathrm{\mu F}\;{\mathrm{cm}}^{-2}$ (Bukoski et al. 2015), the resulting values are presented in Table 1, while the corresponding rate constants are presented in Table 2.

**TABLE 1:** Hodgkin–Huxley model constants demonstrating type‐I behavior.

Symbol	Value
C	1 μF cm^−2^
E_Na_	50 mV
E_K_	−95 mV
E_L_	−63.563 mV
ḡ_Na_	50 mS cm^−2^
ḡ_K_	10 mS cm^−2^
ḡ_L_	0.187 mS cm^−2^
ρ_Na_	60 Channels μm^−2^
ρ_K_	18 Channels μm^−2^
A	30 μm^2^

**TABLE 2:** Voltage‐dependent gating variable rate constants demonstrating type‐I behavior. Units are ms^−1^.

Symbol	Function
α_n_(V)	−0.032(V + 50) / (e^−[(V + 50) / 5]^ − 1)
α_m_(V)	−0.32(V + 52) / (e^−[(V + 52) / 4]^ − 1)
α_h_(V)	0.128e^−[(V + 48) / 18]^
β_n_(V)	0.5e^−[(V + 55) / 40]^
β_m_(V)	0.28(V + 25) / (e^[(V + 25) / 5]^ – 1)
β_h_(V)	4/(e^−[(V + 25) /5]^ + 1)

The membrane area A was chosen relatively small, such that the membrane potential fluctuations amplitude is comparable to that in the experimental time series. Similar results as the ones presented below were obtained for areas up to 10 times the size of the chosen one.

### 2.3 Numerical Simulation

For the numerical simulations, we followed the scheme presented by Chow and White (1996). Instead of keeping track of the state of each individual ion channel, we are only interested in the number of channels in each state. The lifetime of each state at time *t* is given by the following probability density function:

(8) $$f\left(t\right)=\lambda e^{-\lambda \left(t-t_{0}\right)},t\geq t_{0}$$

with

(9) $$\lambda =\sum_{i=0}^{3}\sum_{j=0}^{1}N_{m_{i}h_{j}}\gamma_{\mathrm{ij}}+\sum_{k=0}^{4}N_{n_{k}}\zeta_{k,}$$

where $N_{m_{i}h_{j}}$ is the number of Na^+^ channels in the state $m_{i}h_{j}$ and $\gamma_{\mathrm{ij}}$ is the sum of rate constants associated with escapes from this state, $N_{n_{k}}$ is the number of K^+^ channels in the state $n_{k}$ and $\zeta_{k}$ is the sum of rate constants associated with escapes from this state.

Following this approach, the steps of the algorithm are quite trivial. Starting at the time $t_{0}$ the system is completely described by the membrane potential $V_{0}$ and the number of channels in each state. By knowing the channel state distribution, one can determine the number of Na^+^ and K^+^ channels in the conducting state and thus the Na^+^ and K^+^ conductance.

The first step is to determine when the next transition will occur. This is done by drawing a pseudorandom number $r_{1}$ from the uniform distribution [0,1] and calculating the time of transition by:

(10) $$t_{\mathrm{tr}}=\frac{\ln\left(r_{1}^{-1}\right)}{\lambda }$$

Next, one integrates Equation (4) for the time interval $\mathrm{\Delta t}=t_{\mathrm{tr}}-t_{0}$, using the ion channel distribution at $t_{0}$ to calculate the ion channel conductances, with the assumption that the rate constants do not change during this interval, thus obtaining the value of *V* at the time $t_{1}=t_{0}+t_{\mathrm{tr}}$.

The third step of the algorithm is to determine which one of the possible state transitions took place at the time $t_{\mathrm{tr}}$. The conditional probability that the transition μ took place in the infinitesimally small time interval $\left[t_{0},t_{0}+\mathrm{dt}\right]$ is given by:

(11) $$\frac{\psi_{\mu }\mathrm{dt}}{\sum_{z=1}^{28}\psi_{z}\mathrm{dt}}=\frac{\psi_{\mu }}{\sum_{z=1}^{28}\psi_{z}}$$

where $\psi_{z}\left(1\leq z\leq 28\right)$ is the product of the rate constant characterizing the transition z and the number of channels in the parent state of that transition. Given that the sum in the denominator of (11) is equal to λ:

(12) $$\frac{\psi_{\mu }}{\sum_{z=1}^{28}\psi_{z}}=\frac{\psi_{\mu }}{\lambda }$$

The specific transition μ that occurred is determined by drawing a second pseudorandom number $r_{2}$ from the uniform distribution [0, λ] and calculating the value of μ that satisfies:

(13) $$\sum_{z=1}^{\mu -1}\psi_{z}<r_{2}\leq \sum_{z=1}^{\mu }\psi_{z},\;2\leq \mu \leq 28$$

In the case that $r_{2}\leq \psi_{1}$ it is obvious that μ = 1.

The last step consists of updating the channel states based on the value of μ. One can then start from the beginning, from time $t_{1}$, membrane potential $V_{1}$ and the updated channel state distribution.

Although the aforementioned algorithm has an intrinsic variable timestep, the timeseries were sampled at a rate of 200 kHz while the simulation was running to introduce a constant timestep.

We run simulations for 13 different values of the applied external current density $I_{\mathrm{ex}}$, specifically: $-0.2,-0.1,-0.05,0,0.05,0.1,0.15,0.2,0.25,0.27,0.3,0.32,0.35\;\mathrm{\mu Α}/{\mathrm{cm}}^{2}$ and generated at least three time series of duration greater than 100 s for each value of $I_{\mathrm{ex}}$.

The produced time series were further down‐sampled with a sampling rate of 3125 Hz to match the experimental sampling (Kosmidis et al. 2018), so that the analysis of the time series would correspond to similar timescales and by extension similar generative dynamics of the in vitro neuron. If the sampling is too slow, the signal that is created by the dynamics is lost. Using higher sampling rates leads to an exponential distribution of laminar lengths, indicating an underlying random process, regardless of the value of the applied external current $I_{\mathrm{ex}}$. The reason behind this oversampling effect is that the critical map (1), which is used to approximate the underlying dynamics, provides an effective rather than an exact description of the dynamics. This map describes the dynamics of the system at a coarser timescale, which may consist of several time steps of the underlying Markovian process. Consequently, if the sampling rate is too fast, the recorded signal may not match the timescale at which the critical map (1) can be used as an effective description. In the case, the signal is perceived as mere noise by the critical map, resulting in exponential distributions of the derived laminar lengths.

**FIGURE 2:**
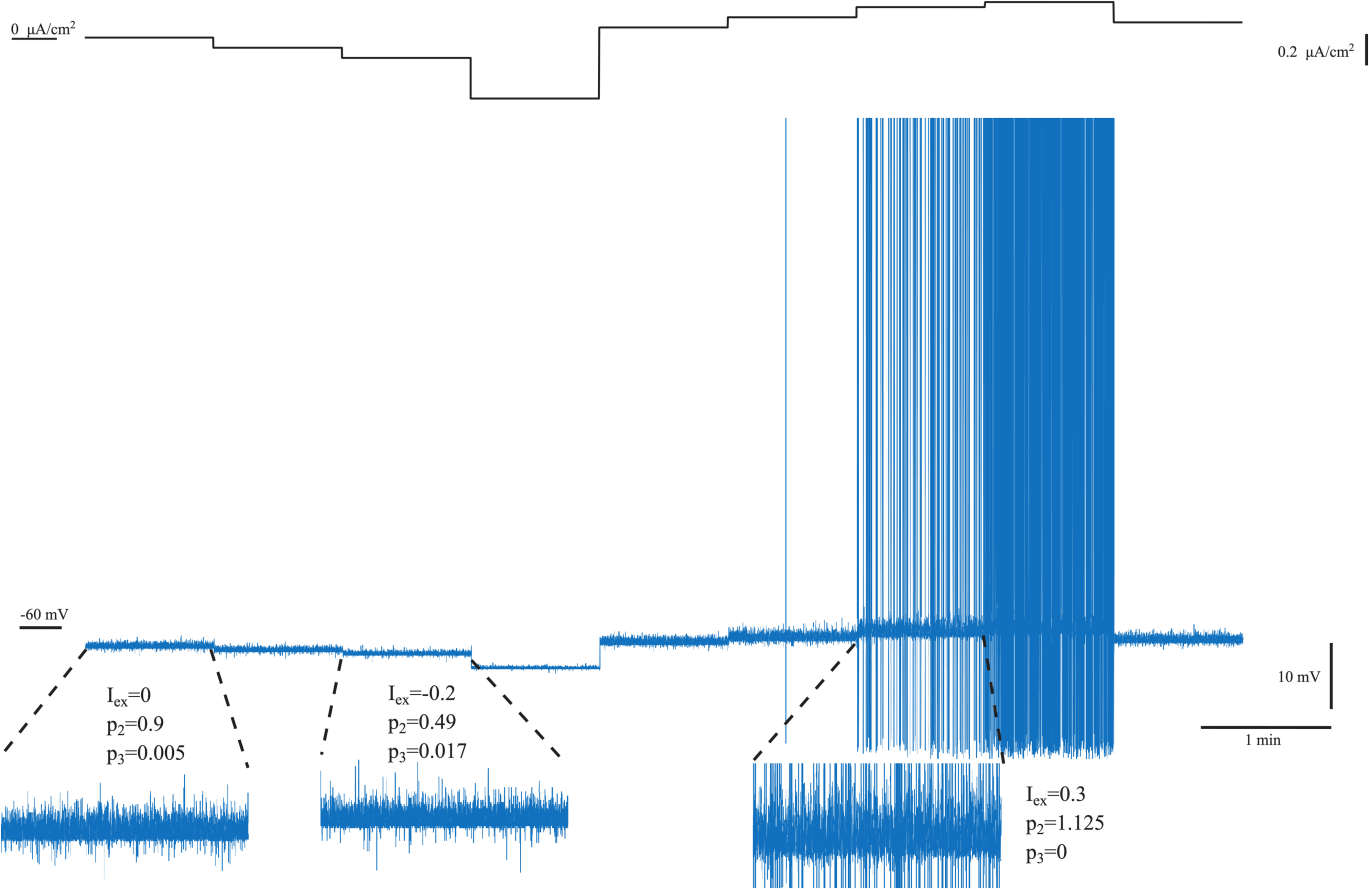
The applied external current density $I_{\mathrm{ex}}$ in $\mathrm{\mu Α}/{\mathrm{cm}}^{2}$ (top) and the corresponding produced membrane potential time series in mV (middle). The subfigures in the lower part show magnified segments of the time series and the corresponding critical exponents acquired from the MCF analysis.

Characteristic segments for different amplitudes of the applied external current density can be seen in Figure 2.

Simulations were implemented using MATLAB R2021a.

## 3 Results

### 3.1 Reproducing the Experimental Results

As it is shown in Figure 2, at rest ($I_{\mathrm{ex}}=0$) there is no firing activity and the membrane fluctuations approach the critical regime, without reaching it ($p_{2}\approx 0.9,p_{3}\approx 0$). Hyperpolarizing the neuron via a negative external current ($I_{\mathrm{ex}}=-0.2$), drives it away from the critical state ($p_{2}=0.49,p_{3}=0.017$), while diminishing the membrane potential fluctuations amplitude. On the other hand, applying a positive stimulation ($I_{\mathrm{ex}}=0.3$) and depolarizing the membrane leads to enhanced critical behavior ($p_{2}=1.125,p_{3}\approx 0$), induces the generation of action potential and amplifies the subthreshold fluctuations.

**FIGURE 3:**
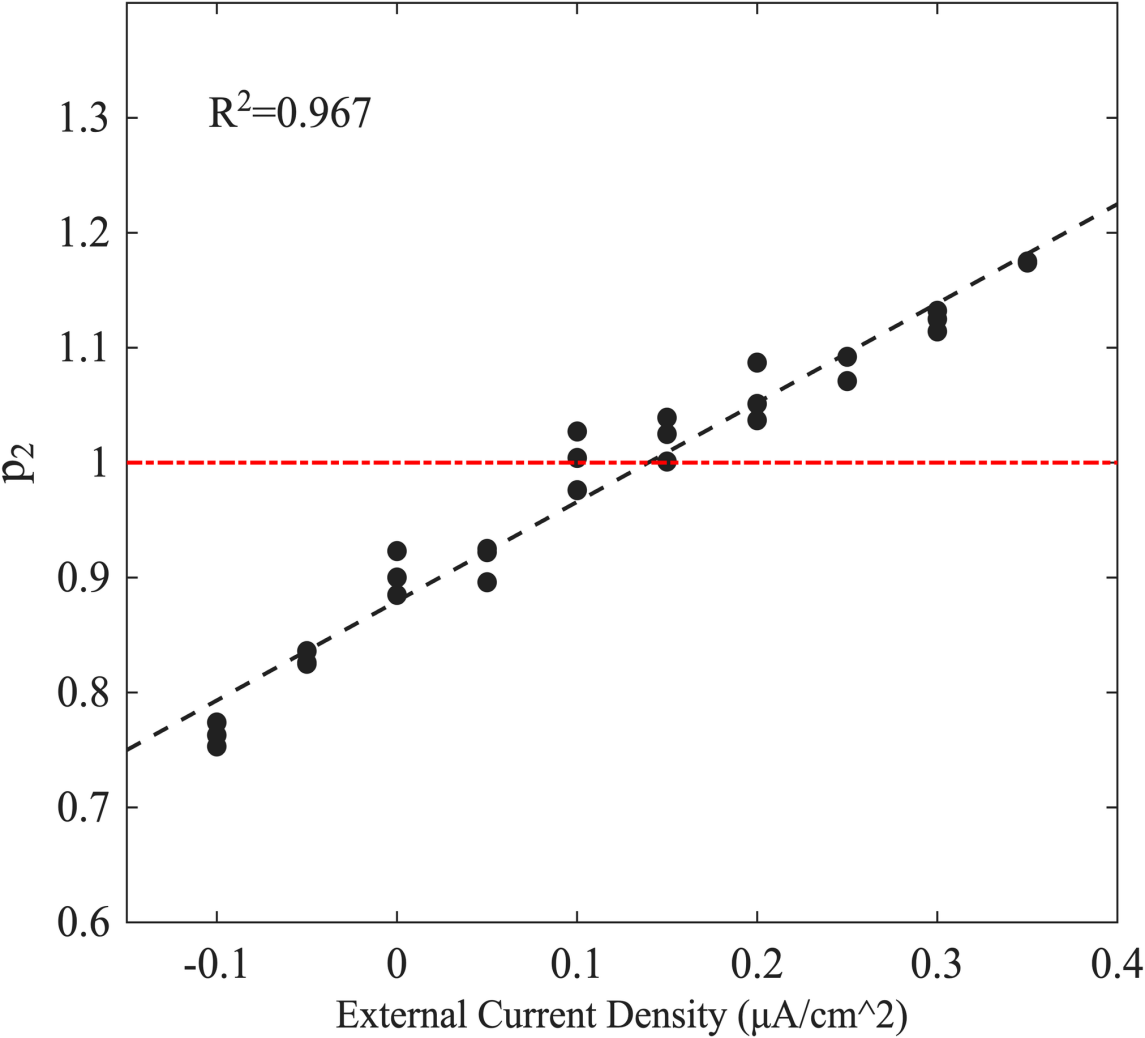
$p_{2}$ exponent as a function of the external current density $I_{\mathrm{ex}}$. Three different time series were analyzed for each value of $I_{\mathrm{ex}}$. The dashed line represents the linear fit.

In total, in segments where the system operates in the critical state or approaches it ($p_{2}\to 1,p_{3}\approx 0$), the relationship between the $p_{2}$ exponent and the amplitude of the external stimulus is almost a linear one as it is demonstrated in Figure 3. This result is in good agreement with the experimental results (Kosmidis et al. 2018). For even more hyperpolarized states, the criticality breakdown is more pronounced, such that $p_{3}$ values become significant, and Equation (3) represents an exponential.

Action potentials cause large deviations in the membrane's voltage but the limits of the laminar region are restricted in the subthreshold regime. Thus, either only the very first part of the action potential plays a role as “laminar length” or the spike starts after the voltage has crossed the upper limit of the laminar region. In the first case, because of its fast growth, only a small number of time steps of the action potential are in the laminar region, resulting in a laminar length of relatively small size. The same goes for the fast‐descending part of the spike. In the second case, it is obvious that the spike does not play a role in forming any laminar lengths. Furthermore, we have analyzed our time series after removing segments of various size centered around spikes, thus keeping only the subthreshold fluctuations. The resulting exponents were identical with the ones described above.

Action potential generation and criticality are found to be highly correlated. Increasing the amplitude of the applied current density $I_{\mathrm{ex}}$ not only results in the amplification of the exponent $p_{2}$ (Figure 3), but also increases the firing rate in the corresponding time series. All of the simulated time series that displayed spiking events, were found to be critical, according to the MCF analysis. On the other hand, segments that were on the margin of being labeled as critical, were not characterized by spiking generation.

**FIGURE 4:**
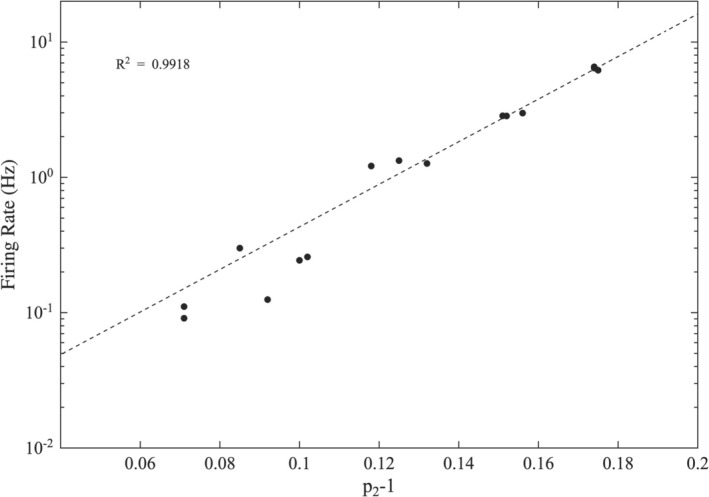
The firing rate as a function of $p_{2}$‐1, calculated from simulated time series with different values of external current density $\left(I_{\mathrm{ex}}\right)$. The dashed line represents an exponential fit.

We can identify a relationship between spiking events and the obtained critical exponents. Since spikes are clearly departures from the laminar region, we can approach them as such. As noted in the Methods section, the laminar region ends when the non‐linear term of the map (1) becomes significant. As the exponent $p_{2}$ increases, $z\to 1^{+}.$ Because the map describes a scaled order parameter $\left(0\leq \Phi \leq 1\right),$ $u\Phi^{z}$ becomes relatively larger as z decreases towards 1. Thus, a larger $p_{2}$ leads to a smaller laminar region, shorter waiting times in that region and consequently a higher spiking rate. If we denote $\bar{\Phi }$, the value for which the linear and the non‐linear terms are equal ($\bar{\Phi }=u{\bar{\Phi }}^{z}$), we obtain the relationship $\ln\bar{\Phi }=\left(p_{2}-1\right)\ln\left(1/u\right)$, where $\ln\bar{\Phi }$ is proportional to the spiking rate. Indeed, in the simulated time series the firing rate and the $p_{2}$ exponent are connected via an exponential relationship (Figure 4), a finding that is in line with the theoretical background and the experimental data (Kosmidis et al. 2018).

### 3.2 The Effect of Noise

In our previous study, we segregated intracellularly recorded membrane potential traces into low‐ and high‐frequency components using cut‐offs between 40 and 100 Hz. High‐frequency components did not carry any information about the system and were best‐fitted by a Gaussian distribution. It was nevertheless necessary for the uncovering of the critical dynamics, since the low‐pass signals alone were not critical, even if they were clearly displaying critical fluctuations before the application of the filtering. Adding artificially generated white noise to the low‐pass signals led to a restoration of the critical exponents (Kosmidis et al. 2018). This stands in line with the finding that the system becomes more ergodic through the addition of uniform noise. This property is essential for the extraction of the critical exponents (Contoyiannis and Diakonos 2007).

**FIGURE 5:**
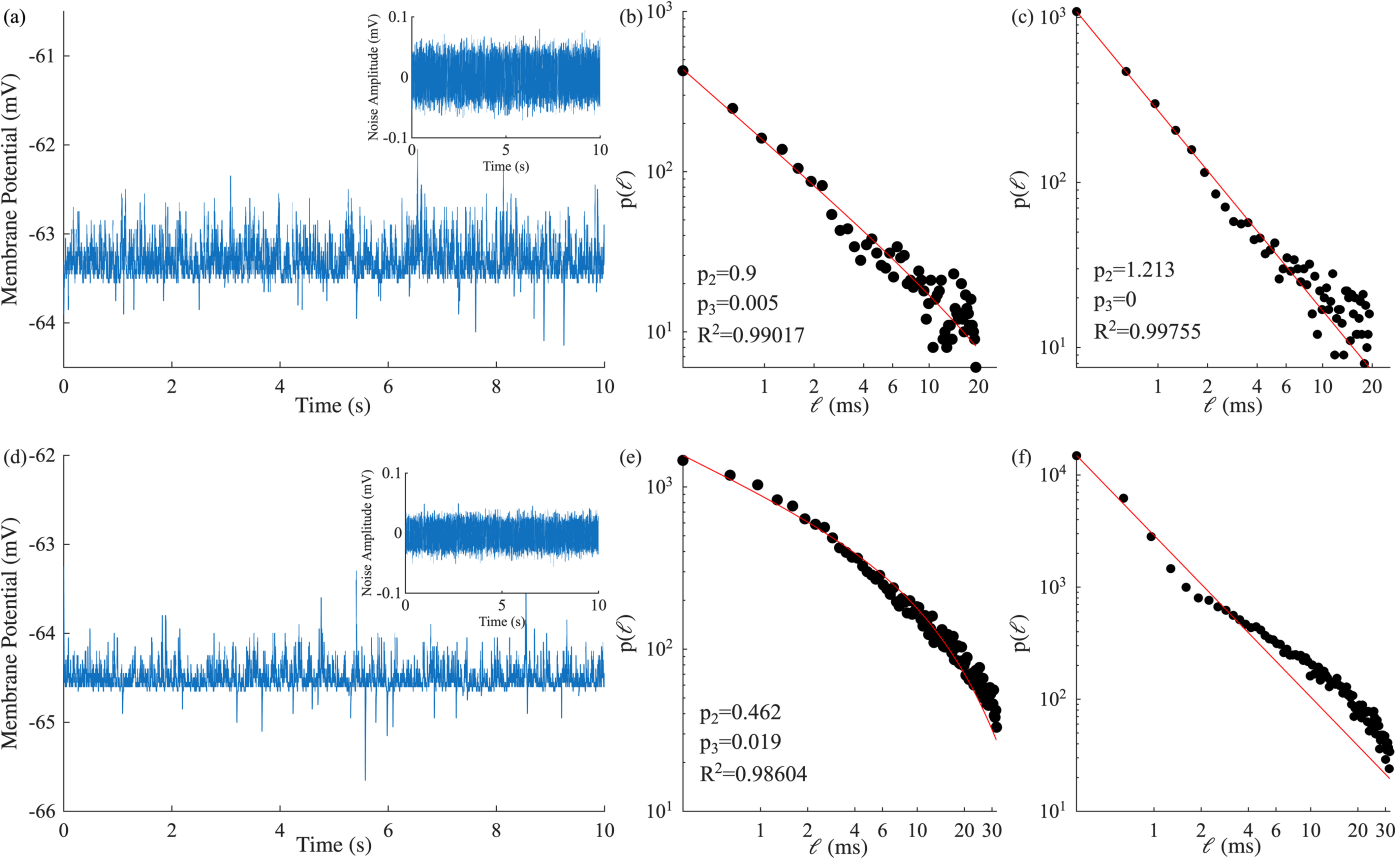
(a) Membrane potential time series segment, with $I_{\mathrm{ex}}=0\;\mathrm{\mu Α}/{\mathrm{cm}}^{2}$. The subplot in the top‐right corner represents the corresponding artificially produced white‐noise. The distribution of laminar lengths $\left(\ell \right)$ with the corresponding critical exponents acquired from the MCF analysis of (a) before (b) ($\phi_{0}=-63.55\;\mathrm{mV},\phi_{R}=-62.9\;\mathrm{mV}$) and after the noise addition (c) ($\phi_{0}=-63.65\;\mathrm{mV},\phi_{R}=-62.8\;\mathrm{mV}$). (d), (e) ($\phi_{0}=-64.7\;\mathrm{mV},\phi_{R}=-64.45\;\mathrm{mV}$), and (f) ($\phi_{0}=-64.7\;\mathrm{mV},\phi_{R}=-64.45\;\mathrm{mV}$) are equivalent subplots for an external current desnity with a value of $I_{\mathrm{ex}}=-0.2\;\mathrm{\mu Α}/{\mathrm{cm}}^{2}$.

For a number of time series with different $I_{\mathrm{ex}}$ we created a normally distributed noise, using a pseudo‐random number generator, with a mean equal to zero and standard deviation of the order of $10^{-1}$ of that of the subthreshold fluctuations of the time series, after removing any existing spikes. Adding this noise to a time series without external stimulation ($I_{\mathrm{ex}}=0$) that was close to criticality but with $p_{2}=0.9<1$ (Figure 5b), lead to a distribution of laminar lengths implying critical behavior with $p_{2}\approx 1.2$ (Figure 5c). Contrary, when white‐noise of this sort was added to a time series with a strong hyperpolarizing current ($I_{\mathrm{ex}}=-0.2$) that was clearly not in a critical regime ($p_{2}=0.462,\;p_{3}=0.019$) (Figure 5d), it resulted in a distribution of laminar lengths that could not be properly fitted by Equation (3) (Figure 5f). In general, the noise addition enhanced the critical dynamics in time series clearly or marginally critical ($I_{\mathrm{ex}}\geq 0$) without generating laminar length distributions that resembled a critical regime in hyperpolarized time series.

The value of $10^{-1}$ was chosen as it is close to the normally distributed noise found in the experimental time series. In general, smaller standard deviations ($<10^{-2}$) either lead to the same results or had little to no effect when added to a time series. Larger standard deviations $\left(>2\times 10^{-1}\right)$ lead to the degradation of the time series and an exponential distribution of laminar lengths, even when added to time series clearly critical beforehand.

### 3.3 Different Ion Contributions and the Effect of K^+^ Channel Blockage

**FIGURE 6:**
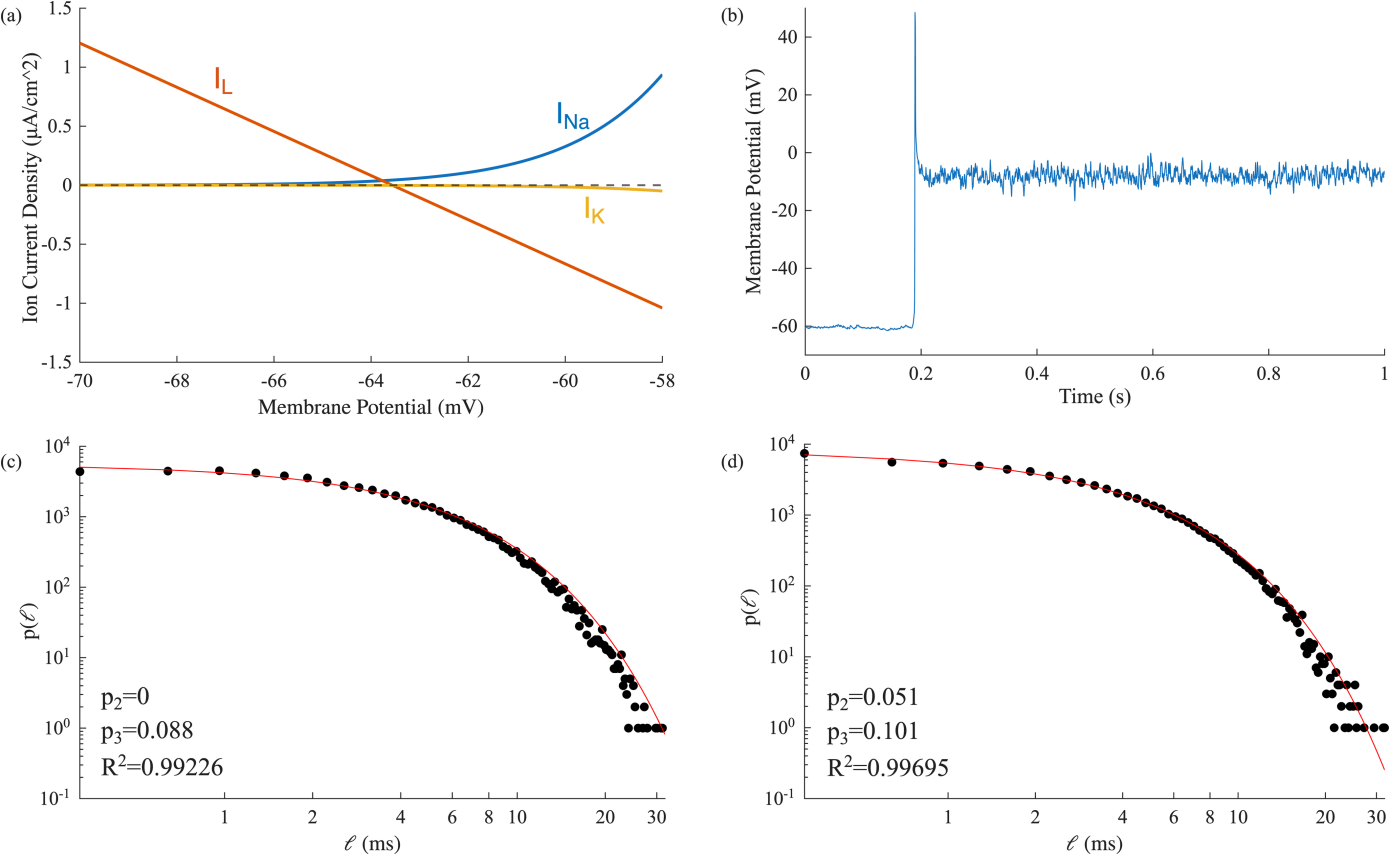
(a) Sodium ($I_{\mathrm{Na}}$), Potassium ($I_{K}$) and Leak ($I_{L}$) ionic currents as a function of the membrane potential, in voltage‐clamped conditions of the deterministic version of our model. (b) Time series segment of the Blocked‐K^+^ model, containing an action potential. Laminar lengths distribution and the result fit of the supra‐threshold fluctuations of the Blocked‐K^+^ model, before (c) ($\phi_{0}=-6\;\mathrm{mV},\phi_{R}=-14\;\mathrm{mV}$) and after (d) ($\phi_{0}=-6\;\mathrm{mV},\phi_{R}=-14\;\mathrm{mV}$) white‐noise addition.

To better estimate the significance of each ion current on the generated dynamics we modeled the deterministic Hodgkin–Huxley model under voltage‐clamped conditions with the present parameterization. In the subthreshold regime of our interest (−65mV<V<−59mV) the contribution of the potassium current, and hence that of the voltage‐dependent potassium channels, is negligible (Figure 6a). We can thus further simplify our system, by considering a model without voltage‐gated potassium channels, where the dynamics are determined solely by the voltage‐gated sodium channels and the leakage current. Indeed, the produced time series and the MCF analysis on the subthreshold regime are almost identical between the two models. This handling can be interpreted in biological terms, as a blocking of the voltage‐dependent potassium channels.

When further depolarized, the blocked‐potassium model gives rise to spike‐like formation. The ascending part is indistinguishable from a regular spike, but because of the lack of a strong repolarizing current, normally generated by the voltage‐dependent potassium channels, the system fluctuates around a new stable point, V≈−7mV (Figure 6b). The membrane potential fluctuations around this new point are much larger, faster and more abrupt than those when the system balances in a subthreshold potential. In this new condition, the criticality is completely lost and the distribution of the laminar lengths represents an exponential fit (Figure 6c). Adding uniform noise following the aforementioned process, did not have any significant effect to the MCF results (Figure 6d).

### 3.4 The Membrane's Bifurcation Point

As a type‐I excitability model, our system undergoes a saddle‐node bifurcation (Izhikevich 2000), when the membrane potential reaches the bifurcation point $V_{c}$. In the infinite system size, $V_{c}$ can be approximated by determining the membrane resting potential for $I_{\mathrm{ex}}=I_{c}$ in the deterministic Hodgkin–Huxley model, where $I_{c}$ is the critical value of the applied current density for spike generation in the deterministic model (Bukoski et al. 2015). For a given time series, the distribution of the membrane potential is restricted around a stationary value $V_{r}$, resulting from the fact that the dynamics tend to a minimization of $\frac{\mathrm{dV}}{\mathrm{dt}}$ (see Equation (4)). $V_{r}$ can be defined as the value of the membrane potential in which the infinite size limit of the system, represented by the deterministic version of the model, stabilizes ($\frac{\mathrm{dV}}{\mathrm{dt}}$ = 0).

**FIGURE 7:**
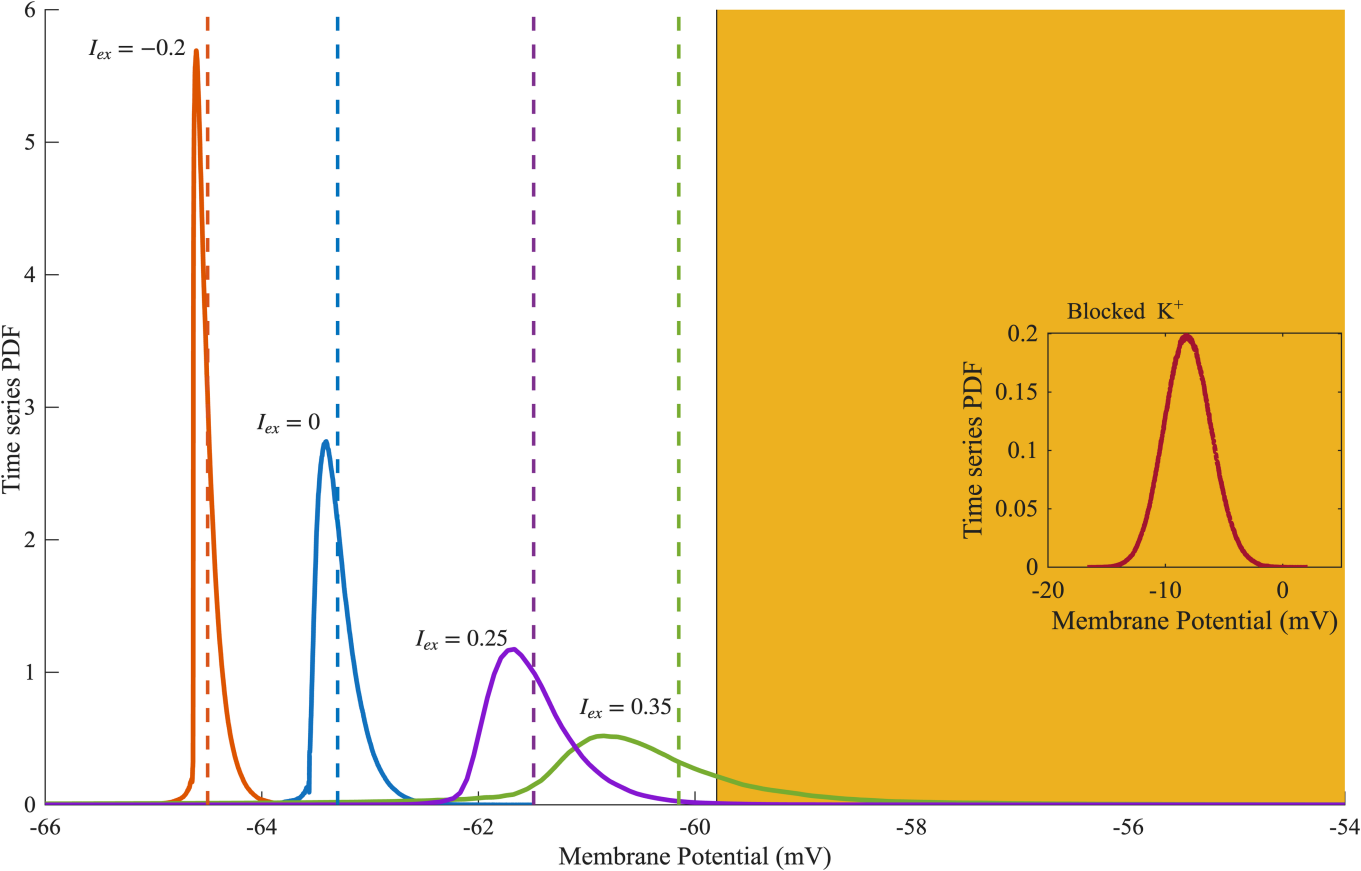
Probability density function histograms of the membrane potential acquired for different values of the external current density ($I_{\mathrm{ex}}$) in $\mathrm{\mu Α}/{\mathrm{cm}}^{2}$. The vertical dashed lines represent the resting potential ($V_{r}$) for the different values of $I_{\mathrm{ex}}$. Displayed on the right is the probability density function histogram of the blocked‐K^+^ model, while it is fluctuating in the supra‐threshold regime. The left (white) part of the figure represents the subthreshold regime, where the right (orange) one represents the suprathreshold regime. In their interface lies the bifurcation point $V_{C}$.

Starting from a membrane potential value $V<V_{c}$ and letting the system evolve in time, we observe that the occasional transition of sodium channels to the conducting state, causes small depolarizations of the membrane, through $I_{\mathrm{Na}}$. This rise to the system's potential, increases in turn the $I_{L}$ leading to a decrease in V. This interplay between $I_{\mathrm{Na}}$ and $I_{L}$ gives rise to the subthreshold fluctuations. If at any time during a fluctuation the membrane potential reaches $V_{c}$, then the system undergoes a phase transition, which is captured by the formation of an action potential. After the transition, the opening of the voltage‐gated potassium channels and thus the increase in $I_{K}$ provides the energy dissipation needed, to restore the system. Depolarizing the membrane via a positive $I_{\mathrm{ex}}$ results in both the shift $V_{r}$ to more positive values, closer to the bifurcation, and of the spreading out of the distribution through the amplification of the fluctuations, a sign of critical slowing‐down, as shown in (Bukoski et al. 2015). Applying a negative $I_{\mathrm{ex}}$ has the opposite effects (Figure 7). Notice how $V_{r}\to V_{c}$, as $I_{\mathrm{ex}}\to I_{c}$. Blocking the voltage‐dependent $K^{+}$ channels results in the pinning of the system in a regime well above the bifurcation point.

## 4 Discussion

### 4.1 The Neuronal Membrane Is Almost Critical

Previous works show that the addition of white‐noise to deterministic versions of the Hodgkin–Huxley model leads to the manifestation of critical characteristics (Steyn‐Ross et al. 2006; Bukoski et al. 2015) and attribute these dynamics to the underlying bifurcation of the model. Nevertheless, adding white‐noise to the deterministic Hodgkin–Huxley does not reproduce our experimentally observed results (Kosmidis et al. 2018). Even though it does produce power‐law distributed characteristics in the membrane potential fluctuations which could in principle hint to criticality, the resulting colored‐noise is unable to reproduce the relationship between the $p_{2}$ exponent and $I_{\mathrm{ex}}$.

Apart from adequately reproducing the experimental findings, implementing the Markov‐chain model allows us to approach the membrane from a complex systems perspective, beyond the classical dynamical approach. Instead of merely solving the differential equations, we treat the membrane as a system consisting of an ensemble of voltage‐gated ion channels. As we mentioned, the major role for the generation of subthreshold fluctuations in the membrane's voltage is played by the sodium and leakage ion channels, while voltage‐gated potassium channels have little to no contribution. When a single sodium channel opens, it creates an inward ionic current, which depolarizes the membrane locally. This local transmembrane voltage gradient gives rise to lateral currents, as described by the cable equation (Hodgkin and Rushton 1946), depolarizing the neighboring membrane patches and forcing the neighboring sodium channels towards the open state. From this perspective, the membrane is a system of ion channels that interact locally with each other, giving rise to the observed membrane potential fluctuations. This draws the picture of a self‐organizing system (Gershenson 2025). Our model is just a mean‐field version of the membrane that neglects the spatial component and averages out the contribution of each channel, as it is based on space‐clamped experimental conditions.

The model exhibits critical intermittency only in the case where the membrane potential fluctuates sufficiently close to the spiking bifurcation. Bias towards the subthreshold or the supra‐threshold regime, via a negative $I_{\mathrm{ex}}$ or the blockage of the potassium channels respectively, abolishes critical behavior. Furthermore, because we observe critical characteristics for a wide range of the parameter region of $I_{\mathrm{ex}}$, rather than a single value, we argue that the system is *almost critical* over this parameter space of $I_{\mathrm{ex}}$ (Kinouchi and Prado 1999). Indeed, it has been claimed that real‐world physical systems, and neuronal systems among them, cannot achieve pure criticality, because they are finite and open (Bonachela et al. 2010). They tend to approach a region, rather than a single critical point, where they display almost critical dynamics and their computational capabilities are optimized, a phenomenon called *quasi‐criticallity* (Williams‐García 2014). Other works provide evidence that cortical networks organize in a marginally subcritical regime, called *reverbarating*, which possesses advantages of both an asychronous and a critical state (Wilting and Priesemann 2019). We hypothesize that a process like self‐organized quasi‐criticality (SOqC) might be the underlying generative mechanism of the observed dynamics (Bonachela and Muñoz 2009), although such an idea requires further exploration.

From an intermittent dynamics perspective, the subthreshold fluctuations resulting from the interplay between sodium and leakage channels give rise to critical intermittency, as indicated from the MCF analysis, while voltage‐gated potassium channels restore the system after a burst occurs, in the form of spike generation. Thus, the dynamics could be interpreted as the result of the coupling of a critical with a tricritical intermittency map, where the former is generated by the sodium and leakage ion channels and the latter from the potassium ion channels (Potirakis et al. 2025).

### 4.2 The Model and Real Neurons

The simulated model exhibits intermittency and operates near a critical point, as it is determined by the MCF analysis. Depolarizing current seems to enhance the critical dynamics, while hyperpolarizing drives the system away from the phase transition. Although these findings are in good agreement with the experimental results (Kosmidis et al. 2018), the acquired critical exponents between the two cases differ. A key difference between experiment and simulation is the state of the neuronal membrane while no external current is being injected into the cell ($I_{\mathrm{ex}}=0$). While most neurons in vitro were found to be critical at rest, this was not the case in our simulated model (Figure 2). Furthermore, the maximal value of the $p_{2}$ exponent was calculated ≈1.18 in the simulated model and ≈1.53±0.13 in the experiment. Adding white noise to the simulated time series resulted in a value of $p_{2}\approx 1.2$ for $I_{\mathrm{ex}}=0$, and rises $p_{2_{\max}}\approx 1.48$, both close to the experimentally estimated values.

It is reasonable to assume that the mismatch between model and experiment can be attributed to experimental noise. Synaptic noise, changes in ion concentrations, neuromodulators, ion pumps, and ephaptic interactions are some of the possible sources of biological noise, besides the stochastic nature of ion channels (Schneidman et al. 1998; Jacobson et al. 2005). Additionally, non‐biological instrumental noise could also be an important noise contributor. These findings lead us to conclude that the signs of criticality in the subthreshold potential fluctuations of the experimentally acquired time series (Kosmidis et al. 2018) can be at least in part attributed to the dynamics of the voltage‐gated ion channels, with the latter being necessary but not sufficient to give rise to the exact dynamics reflected by the values of the acquired critical exponents.

Even if inter‐channel interactions do not reveal the whole picture of the neuronal membrane organization, they might still have important biological implications. While the membrane approaches the spiking bifurcation point, the system's memory, which is encoded in the distribution of the ion channel states, is enhanced and manifests itself in the form of critical slowing down (Bukoski et al. 2015; Meisel et al. 2015). The system is thus able to store and integrate incoming information in a very efficient manner (Haldeman and Beggs 2005). Incoming inputs through chemical or even electrical junctions that further depolarize the cell reinforce the neuron's computational ability.

Our analysis of the reduced mean‐field version of the membrane transforms local interactions between neighboring ion channels via the membrane potential into average whole‐system effects and in that way overlooks the spatial dimension, which is of high significance both in biological neurons and in critical systems. By organizing into a near‐critical state, the membrane could ensure the ability for external perturbations to be transmitted in an efficient way, such that they neither decay too fast nor overwhelm the system (Shew et al. 2011), thus allowing synaptic inputs in the form of EPSPs and IPSPs to efficiently traverse the long distances needed to reach the soma and the hillock.

## 5 Conclusion

Our study shows that an ensemble of ion channels can generate critical intermittency, providing a possible mechanism for the experimentally observed single‐neuron criticality. We suggest that local interactions between sodium channels through the membrane potential play a key role in the emergence of these dynamics through self‐organization. One of our future goals is to model the neuronal membrane in a more rigorous way while preserving the important components of current biophysical models. We hope that future studies will focus on the analysis of the dynamics, including the spatial dimension and the investigation of the effect of various parameters, such as channel density or clustering, Na^+^‐K^+^ pump activity and other ion channel functions, both in computational and experimental settings.

## Author Contributions

**Konstantinos Varvaras:** conceptualization, data curation, formal analysis, investigation, methodology, visualization, writing – original draft, writing – review and editing. **Fotios K. Diakonos:** conceptualization, investigation, methodology, writing – review and editing. **Efstratios K. Kosmidis:** conceptualization, investigation, methodology, supervision, writing – review and editing.

## Conflicts of Interest

The authors declare no conflicts of interest.

## Data Availability

The code used has been deposited in https://github.com/KonVarv/Single‐Neuron‐Criticality.

## References

[ejn70355-bib-0001] Bak, P. 1996. How Nature Works. Spring. 10.1007/978-1-4757-5426-1.

[ejn70355-bib-0002] Bak, P. , C. Tang , and K. Wiesenfeld . 1988. “Self‐Organized Criticality.” Physical Review A 38, no. 1: 364–374. 10.1103/PhysRevA.38.364. 9900174

[ejn70355-bib-0003] Beggs, J. M. , and D. Plenz . 2003. “Neuronal Avalanches in Neocortical Circuits.” Journal of Neuroscience 23, no. 35: 11167–11177. 10.1523/JNEUROSCI.23-35-11167.2003. 14657176 PMC6741045

[ejn70355-bib-0004] Beggs, J. M. , and D. Plenz . 2004. “Neuronal Avalanches Are Diverse and Precise Activity Patterns That Are Stable for Many Hours in Cortical Slice Cultures.” Journal of Neuroscience: The Official Journal of the Society for Neuroscience 24, no. 22: 5216–5229. 10.1523/JNEUROSCI.0540-04.2004. 15175392 PMC6729198

[ejn70355-bib-0005] Bonachela, J. A. , S. de Franciscis , J. J. Torres , and M. A. Muñoz . 2010. “Self‐Organization Without Conservation: Are Neuronal Avalanches Generically Critical?” Journal of Statistical Mechanics: Theory and Experiment 2010, no. 2: P02015. 10.1088/1742-5468/2010/02/P02015.

[ejn70355-bib-0006] Bonachela, J. A. , and M. A. Muñoz . 2009. “Self‐Organization Without Conservation: True or Just Apparent Scale‐Invariance?” Journal of Statistical Mechanics: Theory and Experiment 2009, no. 9: P09009. 10.1088/1742-5468/2009/09/P09009.

[ejn70355-bib-0007] Bukoski, A. , D. A. Steyn‐Ross , and M. L. Steyn‐Ross . 2015. “Channel‐Noise‐Induced Critical Slowing in the Subthreshold Hodgkin‐Huxley Neuron.” Physical Review. E, Statistical, Nonlinear, and Soft Matter Physics 91, no. 3: 032708. 10.1103/PhysRevE.91.032708. 25871145

[ejn70355-bib-0008] Carlson, J. M. , and J. S. Langer . 1989. “Properties of Earthquakes Generated by Fault Dynamics.” Physical Review Letters 62, no. 22: 2632–2635. 10.1103/PhysRevLett.62.2632. 10040041

[ejn70355-bib-0009] Chialvo, R. D. 2004. “Critical Brain Networks.” Physica A: Statistical Mechanics and Its Applications 340, no. 4: 756–765. 10.1016/j.physa.2004.05.064.

[ejn70355-bib-0010] Chow, C. C. , and J. A. White . 1996. “Spontaneous Action Potentials Due to Channel Fluctuations.” Biophysical Journal 71, no. 6: 3013–3021. 10.1016/S0006-3495(96)79494-8. 8968572 PMC1233790

[ejn70355-bib-0011] Christensen, K. , and N. R. Moloney . 2005. Complexity and Criticality. World Scientific Publishing Company.

[ejn70355-bib-0012] Clar, S. , B. Drossel , K. Schenk , and F. Schwabl . 1999. “Self‐Organized Criticality in Forest‐Fire Models.” Physica A: Statistical Mechanics and Its Applications 266, no. 1: 153–159. 10.1016/S0378-4371(98)00587-1.

[ejn70355-bib-0013] Cocchi, L. , L. L. Gollo , A. Zalesky , and M. Breakspear . 2017. “Criticality in the Brain: A Synthesis of Neurobiology, Models and Cognition.” Progress in Neurobiology 158: 132–152. 10.1016/j.pneurobio.2017.07.002. 28734836

[ejn70355-bib-0014] Contoyiannis, Y. F. , and F. K. Diakonos . 2000. “Criticality and Intermittency in the Order Parameter Space.” Physics Letters, Section A 268, no. 4: 286–292. 10.1016/S0375-9601(00)00180-8.

[ejn70355-bib-0015] Contoyiannis, Y. F. , and F. K. Diakonos . 2007. “Unimodal Maps and Order Parameter Fluctuations in the Critical Region.” Physical Review E 76, no. 3: 031138. 10.1103/PhysRevE.76.031138. 17930230

[ejn70355-bib-0016] Contoyiannis, Y. F. , F. K. Diakonos , and A. Malakis . 2002. “Intermittent Dynamics of Critical Fluctuations.” Physical Review Letters 89, no. 3: 035701. 10.1103/PhysRevLett.89.035701. 12144402

[ejn70355-bib-0017] Contoyiannis, Y. F. , F. K. Diakonos , C. Papaefthimiou , and G. Theophilidis . 2004. “Criticality in the Relaxation Phase of a Spontaneously Contracting Atria Isolated From a Frog's Heart.” Physical Review Letters 93, no. 9: 098101. 10.1103/PhysRevLett.93.098101. 15447142

[ejn70355-bib-0018] Diakonos, F. K. , and P. Schmelcher . 1997. “Turning Point Properties as a Method for the Characterization of the Ergodic Dynamics of One‐Dimensional Iterative Maps.” Chaos (Woodbury, N.Y.) 7, no. 2: 239–244. 10.1063/1.166249. 12779652

[ejn70355-bib-0019] Fox, R. F. , and Y. Lu . 1994. “Emergent Collective Behavior in Large Numbers of Globally Coupled Independently Stochastic Ion Channels.” Physical Review E 49, no. 4: 3421–3431. 10.1103/PhysRevE.49.3421. 9961610

[ejn70355-bib-0020] Gal, A. , D. Eytan , A. Wallach , M. Sandler , J. Schiller , and S. Marom . 2010. “Dynamics of Excitability Over Extended Timescales in Cultured Cortical Neurons.” Journal of Neuroscience 30, no. 48: 16332–16342. 10.1523/JNEUROSCI.4859-10.2010. 21123579 PMC6634841

[ejn70355-bib-0021] Gal, A. , and S. Marom . 2013. “Self‐Organized Criticality in Single‐Neuron Excitability.” Physical Review E 88, no. 6: 062717. 10.1103/PhysRevE.88.062717. 24483496

[ejn70355-bib-0022] Gershenson, C. 2025. “Self‐Organizing Systems: What, How, and Why?” Npj Complexity 2, no. 1: 1–8. 10.1038/s44260-025-00031-5.

[ejn70355-bib-0023] Haldeman, C. , and J. M. Beggs . 2005. “Critical Branching Captures Activity in Living Neural Networks and Maximizes the Number of Metastable States.” Physical Review Letters 94, no. 5: 058101. 10.1103/PhysRevLett.94.058101. 15783702

[ejn70355-bib-0024] Herz, A. V. M. , and J. J. Hopfield . 1995. “Earthquake Cycles and Neural Reverberations: Collective Oscillations in Systems With Pulse‐Coupled Threshold Elements.” Physical Review Letters 75, no. 6: 1222–1225. 10.1103/PhysRevLett.75.1222. 10060236

[ejn70355-bib-0025] Hodgkin, A. L. 1948. “The Local Electric Changes Associated With Repetitive Action in a Non‐Medullated Axon.” Journal of Physiology 107, no. 2: 165–181. 10.1113/jphysiol.1948.sp004260. 16991796 PMC1392160

[ejn70355-bib-0026] Hodgkin, A. L. , and A. F. Huxley . 1952. “A Quantitative Description of Membrane Current and Its Application to Conduction and Excitation in Nerve.” Journal of Physiology 117, no. 4: 500–544. 12991237 10.1113/jphysiol.1952.sp004764 PMC1392413

[ejn70355-bib-0027] Hodgkin, A. L. , and W. a. H. Rushton . 1946. “The Electrical Constants of a Crustacean Nerve Fibre.” Proceedings of the Royal Society of London, Series B: Biological Sciences 133, no. 873: 444–479. 10.1098/rspb.1946.0024. 20281590

[ejn70355-bib-0028] Izhikevich, E. M. 2000. “Neural Excitability, Spiking and Bursting.” International Journal of Bifurcation and Chaos 10, no. 6: 1171–1266. 10.1142/S0218127400000840.

[ejn70355-bib-0029] Jacobson, G. A. , K. Diba , A. Yaron‐Jakoubovitch , et al. 2005. “Subthreshold Voltage Noise of Rat Neocortical Pyramidal Neurones.” Journal of Physiology 564, no. 1: 145–160. 10.1113/jphysiol.2004.080903. 15695244 PMC1456039

[ejn70355-bib-0030] Kauffman, S. A. , and S. Johnsen . 1991. “Coevolution to the Edge of Chaos: Coupled Fitness Landscapes, Poised States, and Coevolutionary Avalanches.” Journal of Theoretical Biology 149, no. 4: 467–505. 10.1016/S0022-5193(05)80094-3. 2062105

[ejn70355-bib-0031] Kinouchi, O. , and M. Copelli . 2006. “Optimal Dynamical Range of Excitable Networks at Criticality.” Nature Physics 2, no. 5: 348–351. 10.1038/nphys289.

[ejn70355-bib-0032] Kinouchi, O. , and C. P. C. Prado . 1999. “Robustness of Scale Invariance in Models With Self‐Organized Criticality.” Physical Review E 59, no. 5: 4964–4969. 10.1103/PhysRevE.59.4964. 11969450

[ejn70355-bib-0033] Kosmidis, E. K. , Y. F. Contoyiannis , C. Papatheodoropoulos , and F. K. Diakonos . 2018. “Traits of Criticality in Membrane Potential Fluctuations of Pyramidal Neurons in the CA1 Region of Rat Hippocampus.” European Journal of Neuroscience 48, no. 6: 2343–2353. 10.1111/ejn.14117. 30117214

[ejn70355-bib-0034] Linkenkaer‐Hansen, K. , V. V. Nikouline , J. M. Palva , and R. J. Ilmoniemi . 2001. “Long‐Range Temporal Correlations and Scaling Behavior in Human Brain Oscillations.” Journal of Neuroscience 21, no. 4: 1370–1377. 10.1523/JNEUROSCI.21-04-01370.2001. 11160408 PMC6762238

[ejn70355-bib-0035] Marković, D. , and C. Gros . 2014. “Power Laws and Self‐Organized Criticality in Theory and Nature.” Physics Reports 536, no. 2: 41–74. 10.1016/j.physrep.2013.11.002.

[ejn70355-bib-0036] Meisel, C. , A. Klaus , C. Kuehn , and D. Plenz . 2015. “Critical Slowing Down Governs the Transition to Neuron Spiking.” PLoS Computational Biology 11, no. 2: e1004097. 10.1371/journal.pcbi.1004097. 25706912 PMC4338190

[ejn70355-bib-0037] Nowotny, T. , and M. I. Rabinovich . 2007. “Dynamical Origin of Independent Spiking and Bursting Activity in Neural Microcircuits.” Physical Review Letters 98, no. 12: 128106. 10.1103/PhysRevLett.98.128106. 17501162

[ejn70355-bib-0038] Petermann, T. , T. C. Thiagarajan , M. A. Lebedev , M. A. L. Nicolelis , D. R. Chialvo , and D. Plenz . 2009. “Spontaneous Cortical Activity in Awake Monkeys Composed of Neuronal Avalanches.” Proceedings of the National Academy of Sciences 106, no. 37: 15921–15926. 10.1073/pnas.0904089106. PMC2732708 19717463

[ejn70355-bib-0039] Potirakis, S. M. , F. K. Diakonos , and Y. F. Contoyiannis . 2025. “A Spike Train Production Mechanism Based on Intermittency Dynamics.” Entropy 27, no. 3: 3. 10.3390/e27030267. PMC11941400 40149191

[ejn70355-bib-0040] Ribeiro, T. L. , M. Copelli , F. Caixeta , et al. 2010. “Spike Avalanches Exhibit Universal Dynamics Across the Sleep‐Wake Cycle.” PLoS ONE 5, no. 11: e14129. 10.1371/journal.pone.0014129. 21152422 PMC2994706

[ejn70355-bib-0041] Schneidman, E. , B. Freedman , and I. Segev . 1998. “Ion Channel Stochasticity May Be Critical in Determining the Reliability and Precision of Spike Timing.” Neural Computation 10, no. 7: 1679–1703. 10.1162/089976698300017089. 9744892

[ejn70355-bib-0042] Shew, W. L. , H. Yang , T. Petermann , R. Roy , and D. Plenz . 2009. “Neuronal Avalanches Imply Maximum Dynamic Range in Cortical Networks at Criticality.” Journal of Neuroscience: The Official Journal of the Society for Neuroscience 29, no. 49: 15595–15600. 10.1523/JNEUROSCI.3864-09.2009. 20007483 PMC3862241

[ejn70355-bib-0043] Shew, W. L. , H. Yang , S. Yu , R. Roy , and D. Plenz . 2011. “Information Capacity and Transmission Are Maximized in Balanced Cortical Networks With Neuronal Avalanches.” Journal of Neuroscience: The Official Journal of the Society for Neuroscience 31, no. 1: 55–63. 10.1523/JNEUROSCI.4637-10.2011. 21209189 PMC3082868

[ejn70355-bib-0044] Shriki, O. , J. Alstott , F. Carver , et al. 2013. “Neuronal Avalanches in the Resting MEG of the Human Brain.” Journal of Neuroscience: The Official Journal of the Society for Neuroscience 33, no. 16: 7079–7090. 10.1523/JNEUROSCI.4286-12.2013. 23595765 PMC3665287

[ejn70355-bib-0045] Sneppen, K. , P. Bak , H. Flyvbjerg , and M. H. Jensen . 1995. “Evolution as a Self‐Organized Critical Phenomenon.” Proceedings of the National Academy of Sciences of the United States of America 92, no. 11: 5209–5213. 10.1073/pnas.92.11.5209. 7761475 PMC41878

[ejn70355-bib-0046] Soudry, D. , and R. Meir . 2012. “Conductance‐Based Neuron Models and the Slow Dynamics of Excitability.” Frontiers in Computational Neuroscience 6: 4. 10.3389/fncom.2012.00004. 22355288 PMC3280430

[ejn70355-bib-0047] Stassinopoulos, D. , and P. Bak . 1995. “Democratic Reinforcement: A Principle for Brain Function.” Physical Review E 51, no. 5: 5033–5039. 10.1103/PhysRevE.51.5033. 9963215

[ejn70355-bib-0048] Steyn‐Ross, D. A. , M. L. Steyn‐Ross , M. T. Wilson , and J. W. Sleigh . 2006. “White‐Noise Susceptibility and Critical Slowing in Neurons Near Spiking Threshold.” Physical Review E 74, no. 5: 051920. 10.1103/PhysRevE.74.051920. 17279952

[ejn70355-bib-0049] Stollenwerk, N. 2005. “Criticality in Epidemics: The Mathematics of Sandpiles Explains Uncertainty in Epidemic Outbreaks.” In Recent Advances in Applied Probability, edited by R. Baeza‐Yates , J. Glaz , H. Gzyl , J. Hüsler , and J. L. Palacios , 455–494. Springer US. 10.1007/0-387-23394-6_19.

[ejn70355-bib-0050] Tagliazucchi, E. , P. Balenzuela , D. Fraiman , and D. R. Chialvo . 2012. “Criticality in Large‐Scale Brain fMRI Dynamics Unveiled by a Novel Point Process Analysis.” Frontiers in Physiology 3. 10.3389/fphys.2012.00015. PMC3274757 22347863

[ejn70355-bib-0051] Traub, R. D. , and R. Miles . 1991. Neuronal Networks of the Hippocampus. Cambridge University Press. 10.1017/CBO9780511895401.

[ejn70355-bib-0052] Williams‐García, R. V. 2014. “Quasicritical Brain Dynamics on a Nonequilibrium Widom Line.” Physical Review E 90, no. 6. 10.1103/PhysRevE.90.062714. 25615136

[ejn70355-bib-0053] Wilting, J. , and V. Priesemann . 2019. “Between Perfectly Critical and Fully Irregular: A Reverberating Model Captures and Predicts Cortical Spike Propagation.” Cerebral Cortex 29, no. 6: 2759–2770. 10.1093/cercor/bhz049. 31008508 PMC6519697

